# Macrophage PTEN controls STING-induced inflammation and necroptosis through NICD/NRF2 signaling in APAP-induced liver injury

**DOI:** 10.1186/s12964-023-01175-4

**Published:** 2023-06-27

**Authors:** Tao Yang, Xiaoye Qu, Jiaying Zhao, Xiao Wang, Qian Wang, Jingjing Dai, Chuanlong Zhu, Jun Li, Longfeng Jiang

**Affiliations:** 1 Department of Infectious Diseases, The First Affiliated Hospital With Nanjing Medical University, Nanjing, China; 2 Department of Respiratory and Critical Care Medicine, The Affiliated People’s Hospital of Jiangsu University, The Zhenjiang Clinical Medical College of Nanjing Medical University, Zhenjiang, China; 3 Department of Liver Surgery, Renji Hospital, Shanghai Jiaotong University School of Medicine, Shanghai, China

**Keywords:** PTEN, NICD, NRF2, STING, Macrophages, Liver inflammation

## Abstract

**Background:**

Phosphatase and tensin homolog deleted on chromosome 10 (PTEN) signaling has been known to play a critical role in maintaining cellular and tissue homeostasis, which also has an essential role in the inflammatory response. However, it remains unidentified whether and how the macrophage PTEN may govern the innate immune signaling stimulator of interferon genes (STING) mediated inflammation and hepatocyte necroptosis in APAP-induced liver injury (AILI).

**Methods:**

Myeloid-specific PTEN knockout (PTEN^M−KO^) and floxed PTEN (PTEN^FL/FL^) mice were treated with APAP (400 mg/kg) or PBS. In a parallel in vitro study, bone marrow-derived macrophages (BMMs) were isolated from these conditional knockout mice and transfected with CRISPR/Cas9-mediated Notch1 knockout (KO) or CRISPR/Cas9-mediated STING activation vector followed by LPS (100 ng/ml) stimulation.

**Results:**

Here, we report that myeloid-specific PTEN knockout (PTEN^M−KO^) mice were resistant to oxidative stress-induced hepatocellular injury with reduced macrophage/neutrophil accumulation and proinflammatory mediators in AILI. PTEN^M−KO^ increased the interaction of nuclear Notch intracellular domain (NICD) and nuclear factor (erythroid-derived 2)-like 2 (NRF2) in the macrophage nucleus, reducing reactive oxygen species (ROS) generation. Mechanistically, it is worth noting that macrophage NICD and NRF2 co-localize within the nucleus under inflammatory conditions. Additionally, Notch1 promotes the interaction of immunoglobulin kappa J region (RBPjκ) with NRF2. Disruption of the Notch1 signal in PTEN deletion macrophages, reduced RBPjκ and NRF2 binding, and activated STING signaling. Moreover, PTEN^M−KO^ macrophages with STING activated led to ROS generation and TNF-α release, resulting in hepatocyte necroptosis upon co-culture with primary hepatocytes.

**Conclusions:**

Our findings demonstrate that the macrophage PTEN-NICD/NRF2-STING axis is critical to regulating oxidative stress-induced liver inflammation and necroptosis in AILI and implies the therapeutic potential for managing sterile liver inflammation.

Video Abstract

**Graphical Abstract:**

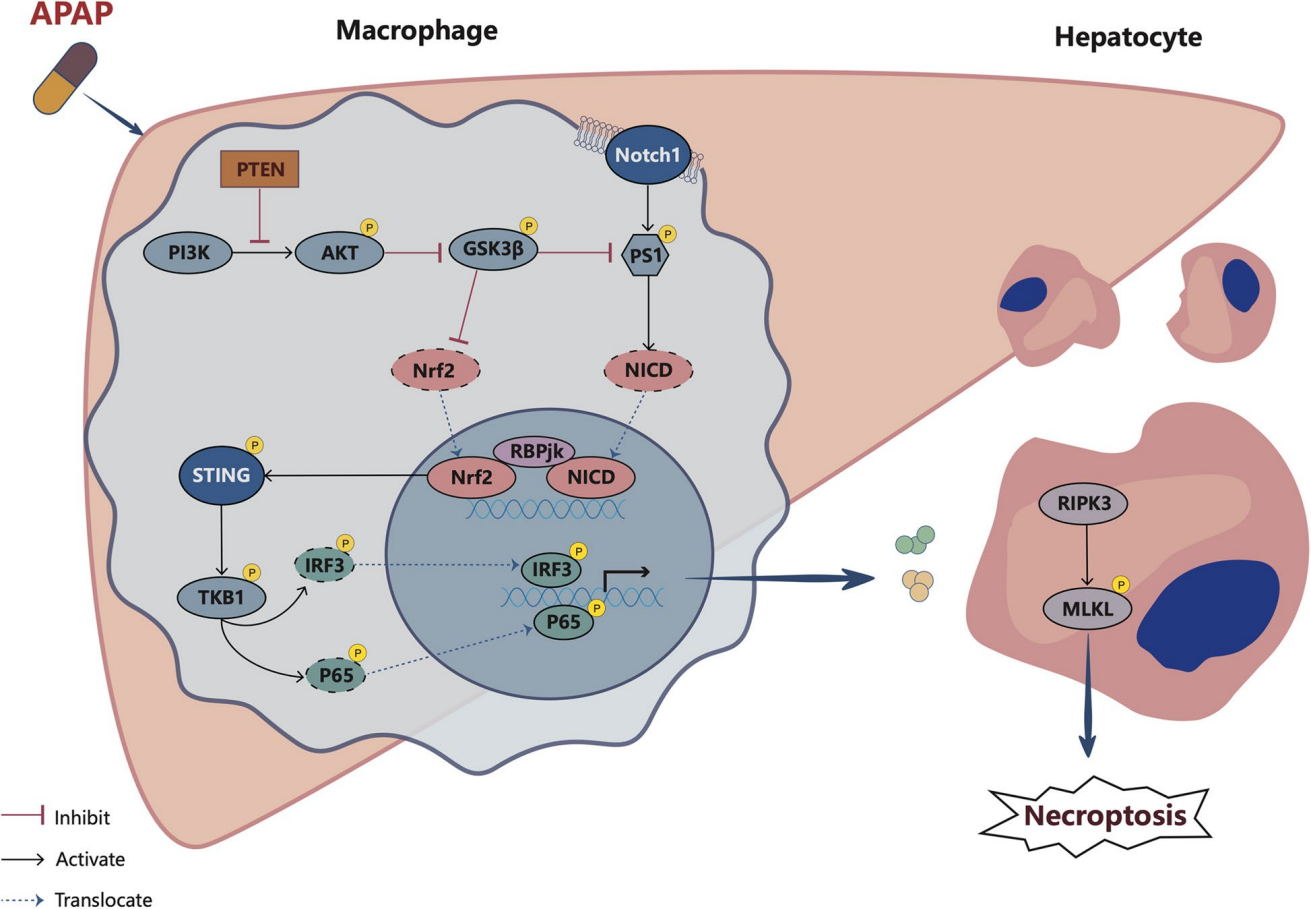

**Supplementary Information:**

The online version contains supplementary material available at 10.1186/s12964-023-01175-4.

## Introduction

Acetaminophen (N-acetyl-para-aminophenol) (APAP) hepatotoxicity with an overdose can lead to severe hepatic damage, ultimately resulting in acute liver failure (ALF) [1, 2]. The survival improvement for progressive ALF is needed for liver transplantation [3]. The APAP-induced liver injury (AILI) is affected by the dosage of APAP for the initial hepatocyte injury and the inflammatory response, which is identified by activating hepatic macrophages and neutrophils, according to mouse liver injury models and pertinent clinical research. This activation leads to the release of pro-inflammatory cytokines/chemokines and exacerbates ROS generation and hepatocyte death [4, 5].

Phosphatase and tensin homolog deleted on chromosome 10 (PTEN) has been known to play a critical role during the inflammatory response [6]. PTEN is a crucial regulator of phosphoinositide3-kinase (PI3K)/Akt prosurvival signaling by antagonizing PI3K to convert PtdIns(3,4,5)P_3_ to PtdIns(4,5)P_2_ [7, 8]. Increasing PTEN activity aggravates tissue inflammation, and deletion of PTEN activates PI3K/Akt signaling and alleviates inflammatory response [9]. Moreover, PI3K/Akt activation by PTEN inhibition protects against ischemic heart failure and brain injury [10, 11]. The previous study has demonstrated that PTEN/PI3K regulates innate Toll-like receptor (TLR)4-driven inflammatory response in mouse liver ischemia–reperfusion injury [12]. Although it has been demonstrated that deletion of PTEN signaling is related to hepatocellular protection in liver inflammation, more has to be understood about the underlying mechanisms involving PTEN signaling and hepatic inflammatory responses.

Notch is a cell membrane receptor that plays a crucial role in regulating cell proliferation, differentiation, and development [13]. Studies have indicated that Notch signaling in mammalian cells includes five ligands (Delta-like 1 (DLL1), DLL3, and DLL4, Jagged1, Jagged2), and four receptors (Notch1-4) [14]. Upon activation by ligands, Notch can be cleaved by disintegrin, metalloprotease family proteases, and γ-secretase that form the Notch intracellular structural domain (NICD). The NICD is then translocated to the nucleus and binds to the nuclear co-transcription factor recombination signal binding protein for the immunoglobulin kappa J region (RBPjκ). This interaction converts RBPjκ from a transcriptional repressor to an activator, eventually activating TLR4-MYD44, JNK-P65, and other signaling pathways to control cell death [15]. In our previous study, we discovered that Notch1 signaling controls the HMGB1/TLR4/NF-κB axis in AILI to regulate NLRP3 inflammasome activation, illuminating the critical function of the Notch1-Hes1 signaling cascade response in regulating innate immunity in APAP-triggered liver inflammation [16]. Our recent research has also revealed the vital role of the macrophage Notch1-β-catenin axis as a regulatory mechanism in ischemia–reperfusion related liver inflammation[17]. However, it is still understood how PTEN and Notch1 crosstalk in AILI needs to be further studied.

It has been acknowledged that the stimulator of interferon genes (STING) pathway is an essential signaling pathway in the innate immune system [18], which is highly expressed in hepatic macrophages and is not measurable in human and murine hepatocytes [19, 20]. While myeloid STING disruption reduced hepatic inflammation in non-alcoholic fatty liver disease, STING augmented macrophage-mediated inflammatory responses and impaired liver function [19, 20]. A previous study has indicated that the transcription factors NF-κB and interferon (IFN) regulatory factor (IRF) 3 were activated by STING through the TANK-binding kinase 1 (TBK1) [21]. Whereas how PTEN drives STING signaling-mediated programmed death needs to be further investigated.

In the current study, we substantiated the unique regulatory function of macrophage PTEN in AILI. We demonstrated a novel mechanism of macrophage PTEN deficiency that regulates nuclear factor (erythroid-derived 2)-like 2 (NRF2) function, interrelates with NICD, and then governs STING-mediated TBK1 function, resulting in amelioration of inflammatory responses and reducing APAP-triggered hepatotoxicity. We have unearthed that the macrophage PTEN-NICD/NRF2-STING cascade is crucial in fine-tuning the innate immune responses in the pathogenesis of AILI.

## Materials and methods

### Animals

Floxed PTEN (PTEN^FL/FL^) mice (The Jackson Laboratory, Bar Harbor, ME) and the mice expressing the Cre recombinase under the control of the Lysozyme M (LysM) promoter (LysM-Cre; The Jackson Laboratory) were used to generate myeloid-specific PTEN knockout (PTEN^M−KO^) mice, as described [22]. All animals were age- and sex-matched and housed in an animal facility under specific pathogen-free conditions. The laboratory animals were maintained on a consistent environmental regimen, which encompassed 12-h light–dark cycles and a controlled diet comprising water and food. All animal studies were approved by the Institutional Animal Care and Use Committee (IACUC) of Nanjing Medical University, China.

### Mouse model and treatment

Acetaminophen (APAP, Sigma-Aldrich) solution configuration as previously described [16]. For each experiment, the APAP dissolved in phosphate-buffered saline (PBS) at a concentration of 10 mg/ml and warmed to 40℃. Mice were received either 400 mg/kg of APAP or PBS intraperitoneally (i.p.) after fasting for 16 h. In some experiments, mice have been injected with the nonspecific (NS) control siRNA or Nrf2 siRNA (2 mg/kg; Santa Cruz Biotechnology (SC), sc-37049) mixed with mannose-conjugated polymers (Polyplus transfection™, Illkirch, France) at a ratio via the tail vein 24 h before APAP injection, according to the manufacturer’s instructions, as previously described [23]. Animals were euthanized by ketamine/xylazine injection at the indicated time-point for collecting serum and liver tissues. Animal survival was observed every 4 h for 72 h until they became moribund.

### Hepatocellular function assay

Serum alanine aminotransferase (sALT) and aspartate aminotransferase(sALT) levels were detected by ALT and AST kit (ThermoFisher, Waltham, MA) according to the manufacturer’s instructions.

### Histology, immunohistochemistry and immunofluorescence staining

Liver tissue was harvested and rinsed with PBS and then immersed into 10% of buffered formalin overnight. After processing for paraffin embedding, the liver Sects. (5 μm) were stained with hematoxylin and eosin (H&E). Suzuki’s criteria were used to grade the severity of liver damage [24].

The immunohistochemistry (IHC) staining was used consecutive 5 μm thick sections from the liver tissue. The sections were deparaffinized and hydrated through xylene and graded alcohols, processed for an antigen-unmasking procedure, then pretreated and stained with antibodies with the Ready-to-use IHC kit (BioVision, 405–50). The neutrophils were detected using primary Ly6G mAb (Invitrogen, 14–5931-82). The necroptosis was detected using primary RIPK3 mAb (Cell Signaling Technology (CST),10188).

The 5 μm thick frozen sections were thaw at room temperature for 10–20 min, then washed with PBST. The sections and cell slides were fixed with formalin for 15 min and incubated in blocking buffer (5% BAS) for 1 h. The primary antibodies were incubated overnight at 4℃. Then add a secondary antibody and incubated for 1 h. Rinsed the slides 5–7 times with PBST. DAPI was used for nuclear counterstaining. Liver macrophages were detected using primary rat anti-mouse F4/80 mAb (SC, sc-377009). The primary rabbit PTEN (Invitrogen, PA5-1554), p-GSK3β (CST, 5558), and NRF2 (CST, 12721) mAbs, primary mouse CD68 (SC, sc-17832) and NICD (SC, sc-373891) mAbs were used for the double immunofluorescence staining according to the manufacturer’s instructions. Hepatocyte p-MLKL (mixed lineage kinase domain-like) was detected using primary p-MLKL rabbit mAb (CST, 37333). The secondary AlexFluor488- conjugated AffiniPure donkey anti-rabbit IgG Ab (711–545-152), AlexFluor488- conjugated AffiniPure donkey anti-mouse IgG Ab (715–545-150), Cy™5 AffiniPure Donkey Anti-rabbit IgG Ab (711–175-152), Cy™5 AffiniPure Donkey Anti-Mouse IgG (715–175-150) (Jackson Immunoresearch). Images for immunofluorescence staining were captured using a fluorescence microscope (LEICA DMI3000B) and analyzed using Image-J software. Positive cells were counted blindly in 10 HPF/section (× 200 magnification).

### ELISA assay

The cell culture supernatants were harvested for cytokine analysis. The ELISA kit (ThermoFisher Scientific) was used to measure TNF-α levels according to the manufacturer’s instructions.

### LDH activity assay

BMMs (1 × 10^6^) were cocultured with primary hepatocytes (4 × 10^5^ /well) for 12 h with or without adding H_2_O_2_ (200 µM) in the lower chamber. The activity of lactate dehydrogenase (LDH) in the cell culture medium from the lower chamber was measured with a commercial LDH activity assay kit (Stanbio Laboratory, Boerne, TX) according to the manufacturer’s instructions.

### Western blot analysis

Protein was extracted from liver tissue or cell cultures as described [25]. The nuclear and cytosolic fractions were prepared with NE-PER Nuclear and Cytoplasmic Extraction Reagents Kit (ThermoFisher Scientific). Proteins (30 μg/sample) were subjected to 12% SDS–polyacrylamide gel electrophoresis and transferred to a nitrocellulose membrane (Bio-Rad, Hercules, CA). Monoclonal anti-rabbit PTEN (9188), Akt (4691), p-Akt (Ser473) (4060), GSK3β (12456), p-GSK3β (Ser9) (5558), NICD (3608), NRF2 (12721), STING (13647), p-STING (Ser366) (50907), TBK1 (38066), p-TBK1 (Ser172) (5483), IRF3 (11904), p-IRF3 (Ser396) (29047), P65 (8242), p-P65 (Ser356) (3033), RIPK3 (10188), p-MLKL(Ser345) (37333), Lamin B2 (24209), β-actin Abs (12262) (CST), p-Presenilin 1/PS-1 (Ser357) (ab78914), Presenilin 1/PS-1 (ab76083) and RBPjκ (ab25949) (Abcam) were used to overnight incubation at 4℃. Then add a secondary antibody solution and incubate for 1 h. Rinse the blot 5–7 times with TBST. The Western ECL substrate mixture (Bio-Rad) was used to image with the iBright FL1000 (ThermoFisher Scientific).

### Quantitative RT-PCR analysis

The RT-PCR was performed as described [26]. Total RNA was purified from liver tissue or cell cultures using RNeasy Mini Kit (Qiagen, Chatsworth, CA) according to the manufacturer’s instructions. The total RNA (2.5 μg) was reverse-transcribed into cDNA using the SuperScript™ III System (Invitrogen, CA). Quantitative real-time PCR was carried out using the QuantStudio 3 (Applied Biosystems by ThermoFisher Scientific). The following were added in the final reaction volume of 25 μl: 1 × SuperMix (Platinum SYBR Green qPCR Kit; Invitrogen) cDNA and 10 μM of each primer. The amplification conditions were 50 °C (2 min), 95 °C (5 min), followed by 40 cycles of 95 °C (15 s) and 60 °C (30 s). Primer sequences used to amplify *Tnf-α*, *IL-1β*, *IL-6*, *Mcp1*, *Cxcl-1*, *Inf-β, Nqo1*, *Gclc*, and *Gclm* are shown in Table S1. Target gene expressions were calculated by their ratios to the housekeeping gene β-actin.

### Reactive oxygen species assay

ROS production in Kupffer cells was measured using the 5-(and-6)-carboxy-2',7'-difluoro dihydro fluorescein diacetate (Carboxy-H2DFFDA, ThermoFisher Scientific) according to the manufacturer’s instructions. In brief, Macrophages were cultured on collagen-coated coverslips after LPS stimulation. After washing with PBS, cells were then incubated with 10 μM of Carboxy-H2DFFDA. The Carboxy-H2DFFDA was converted to a green-fluorescent form when hydrolyzed by intracellular esterase and oxidized in the cells. ROS produced by Macrophages were analyzed by fluorescence microscopy. Positive green fluorescent-labeled cells were counted blindly in 10 HPF/section (× 200).

### Isolation of primary hepatocytes, liver macrophages, and bone marrow-derived macrophages in vitro transfection

Primary hepatocytes, liver macrophages (Kupffer cells), and bone marrow-derived macrophages (BMMs) from the PTEN^FL/FL^, PTEN^M−KO^, or wild-type (WT) mice were isolated as described [27]. Briefly, livers were perfused in situ with warmed (37℃) EGTA-containing (Sigma-Aldrich, 0.5 mmol/L) HBSS solution for 5 min, followed by a collagenase buffer (collagenase type IV, Sigma-Aldrich, 100CDU/ml) for 5 min until liver digestion was visible. Perfused livers were dissected and teased through 70-μm nylon mesh cell strainers (BD Biosciences, San Jose, CA). Nonparenchymal cells (NPCs) were separated from hepatocytes by centrifuging at 50 × g for 2 min three times. NPCs were suspended in HBSS and layered onto a 50%/25% two-step Percoll gradient (Sigma) in a 50-ml conical centrifuge tube and centrifuged at 1800 × g at 4 °C for 15 min. Kupffer cells in the middle layer were collected and attached to cell culture plates in DMEM with 10% FBS, 10 mM HEPES, 2 mM GlutaMax, 100 U/ml penicillin, and 100 μg/ml streptomycin for 15 min at 37 °C. Nonadherent cells were removed by replacing the culture medium. Bone marrow cells were removed from the femurs and tibias of PTEN^FL/FL^ and PTEN^M−KO^ mice cultured in Dulbecco’s Modified Eagle’s Medium (DMEM) supplemented with 10% FBS and 15% L929-conditioned medium. BMMs (1 × 10^6^/well) cultured for 7 days were transfected with CRISPR/Cas9 Notch1 knockout (KO) (sc-421930-KO-2), CRISPR/Cas9 Tmem173 (STING) knockout (KO) (sc-428364), CRISPR-Tmem173 activation (sc-428364-ACT) or control vector (Santa Cruz Biotechnology) according to the manufacturer’s instructions. After 48 h, the cells were supplemented with 100 ng/ml of LPS for an additional 6 h.

### Co-culture of macrophages and primary hepatocytes

The co-culture system was conducted as described [27]. In brief, primary hepatocytes were cultured in 6-well plates at a concentration of 4 × 10^5^ cells per well. After 24 h, the 0.4 μm-pore size transwell inserts (Corning) containing 1 × 10^6^ BMMs were placed into the 6-well plate with the initially seeded hepatocytes. Co-cultures were incubated for 12 h with adding H_2_O_2_ (200 µM) in the lower chamber.

### Immunoprecipitation analysis

BMMs from co-culture were lysed in NP-40 lysis buffer (50 mM Tris pH 7.4, 10 mM EDTA, 150 mM NaCl, and 1% NP-40, ThermoFisher Scientific) containing protease inhibitors. The cell lysates were pre-cleared with protein A/G beads at 4 °C for 2 h with rotation, then incubated with primary antibody or control IgG at 4 °C overnight. The cell lysates were then incubated with protein A/G beads for an additional 2 h. Following the process of immunoprecipitation, the immunocomplexes were washed with lysis buffer three times and analyzed by standard immunoblot procedures.

### Statistical analysis

Data are expressed as mean ± SD and analyzed by using the permutation t-test and Pearson correlation. Multiple group comparisons were performed using one-way ANOVA with a post hoc test. Per comparison two-sided p values less than 0.05 were considered statistically significant. All analysis was performed in Prism 9 (GraphPad Software, version 9.5.0).

## Results

### Macrophage PTEN deficiency alleviates APAP-induced liver injury (AILI) and decreases macrophage/neutrophil infiltration

**Fig. 1:**
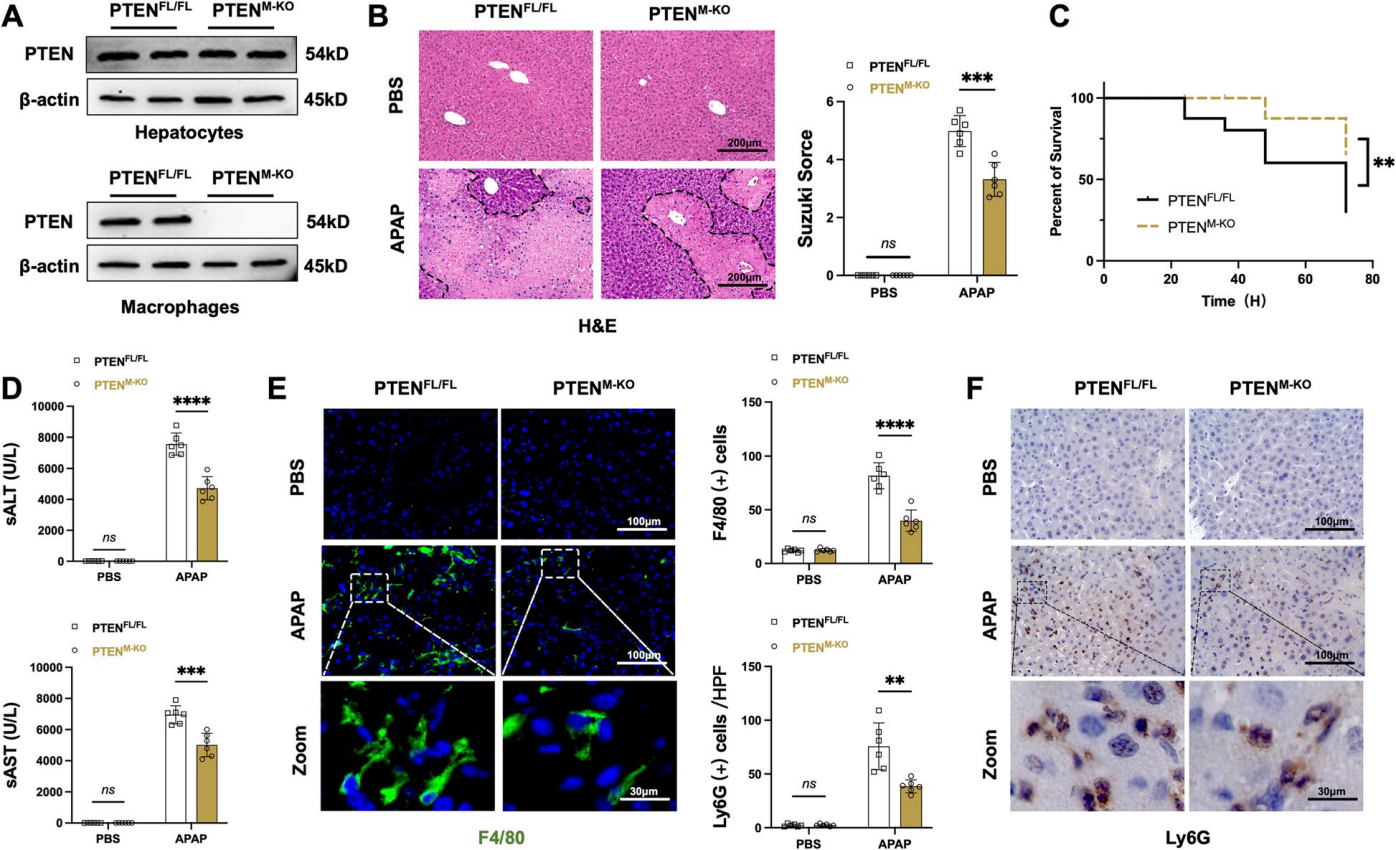
Macrophage PTEN deficiency alleviates APAP-induced liver injury and decreases macrophage/neutrophil infiltration. Mice were subjected to APAP (400 mg/kg) challenge for 24 h. **A** Western blot assay detected the PTEN expression in hepatocytes and liver macrophages from PTEN^FL/FL^ and PTEN^M−KO^. Representative of three experiments. **B** Representative histological staining (H&E) of APAP-conditioning liver tissue and Suzuki’s histological score (*n* = 6 samples/group). Scale bars, 200 μm. **C** Animal survival curves following a solitary APAP dose administered over 72 h (*n* = 6 mice/group). **D** Hepatocellular function, assessed by serum ALT (sALT) and AST (sALT) levels (IU/L) (*n* = 6 samples/group). **E** Immunofluorescence staining of F4/80 macrophages in injury livers (*n* = 6 mice/group). Quantification of F4/80^+^ macrophages. Scale bars, 100 μm and 30 μm. **F** Immunohistochemistry staining of Ly6G neutrophils in injury livers (*n* = 6 mice/group). Quantification of Ly6G^+^ neutrophils. Scale bars, 100 and 30 μm. All data represent the mean ± SD. **p < 0.01, ***p < 0.001, ****p < 0.0001

To test whether myeloid-specific PTEN ablation may affect inflammatory responses in mouse livers, we used a mouse model of AILI. The primary hepatocytes and liver macrophages were isolated from these models. In contrast to the PTEN^FL/FL^ mouse model, PTEN^M−KO^ exhibited a deficiency of PTEN expression solely in liver macrophages rather than hepatocytes (Fig. 1A). The hepatocellular damage was evaluated in mouse livers 24 h after the APAP challenge. Livers in PTEN^M−KO^ mice showed less hepatic hemorrhage and necroinflammation after APAP treatment. However, the PTEN^FL/FL^ liver displayed more hepatic hemorrhage and severe necrosis (Fig. 1B). The liver damage assessment was conducted by implementing Suzuki's histological grading for liver injury (Fig. 1B). To determine whether macrophage PTEN deficiency may affect animal survival, APAP-challenged mice were monitored for 72 h. The mortality in PTEN^M−KO^ mice significantly declined compared to the PTEN^FL/FL^ controls after APAP treatment (Fig. 1C). Consistent with the histology data, the serum ALT (sALT) and AST (sAST) levels (U/L) in PTEN^M−KO^ mice significantly declined compared to the PTEN^FL/FL^ controls. Moreover, the PTEN^M−KO^ livers showed reduced accumulation of F4/80^+^ macrophages (Fig. 1E) and Ly6G^+^ neutrophils (Fig. 1F). These results demonstrate that macrophage PTEN deficiency plays an important role in preventing AILI and inflammatory responses.

### Macrophage PTEN deficiency inhibits STING-mediated TBK1 activation and diminishes APAP-induced hepatocyte death

**Fig. 2:**
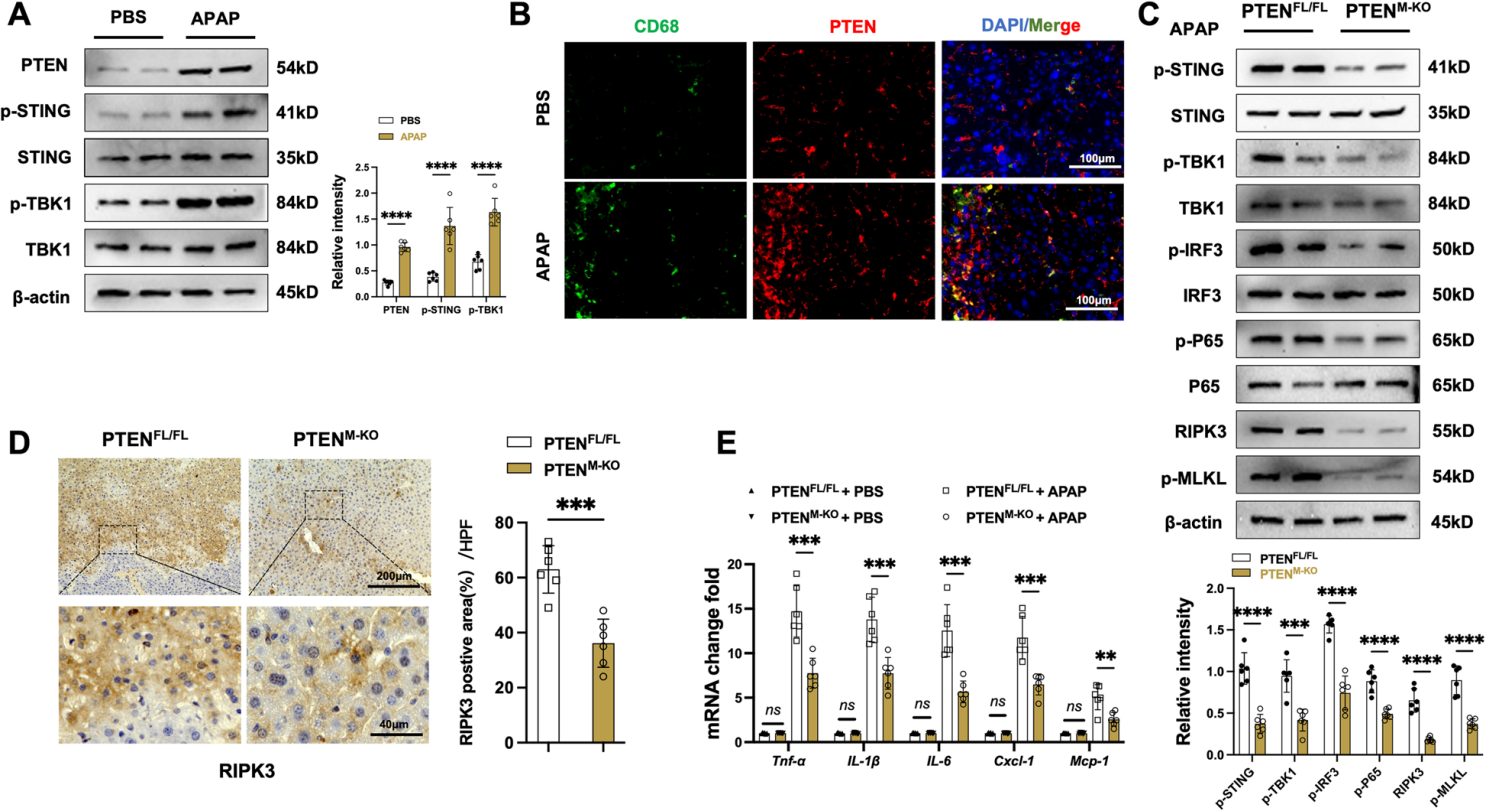
Macrophage PTEN deficiency inhibits STING-mediated TBK1 activation and diminishes APAP-induced hepatocyte death. PTEN^FL/FL^ and PTEN^M−KO^ mice were subjected to APAP (400 mg/kg) challenge for 24 h. **A** Western-assisted analysis and relative density ratio of PTEN, p-STING, STING, p-TBK1, and TBK1 in the PTEN^FL/FL^ livers with or without APAP-treated (*n* = 6 samples/group). **B** Immunofluorescence staining of PTEN and CD68 in AILI livers (*n* = 6 mice/group). Scale bars, 100 μm. **C** Western-assisted analysis and relative density ratio of p-STING, STING, p-TBK1, TBK1, p-IRF3, IRF3, p-P65, P65, RIPK3, and p-MLKL in the PTEN^FL/FL^ and PTEN^M−KO^ mice livers after APAP challenged (*n* = 6 samples/group). **D** Immunohistochemistry staining of RIPK3 expression in APAP-challenged livers (*n* = 6 mice/ group). Scale bars, 200 and 40 μm. **E** Quantitative RT-PCR-assisted detection of *Tnf-α*, *IL-1β*, *IL-6*, *Cxcl-1,* and *Mcp-1* mRNA levels in PTEN^FL/FL^ and PTEN^M−KO^ mouse livers with or without APAP exposure (*n* = 6 samples/group). All data represent the mean ± SD. **p < 0.01, ***p < 0.001, ****p < 0.0001

To determine whether PTEN activation is associated with APAP challenges, we first detect the PTEN expression in the liver after APAP treatment. As expected, the PTEN expression was upregulated in APAP-challenged livers compared to the controls (Fig. 2A). Moreover, APAP-challenged augmented phosphorylation of STING and TBK1 has been observed in the liver (Fig. 2A). Subsequently, using CD68 as a marker for hepatic macrophages [28], we assessed PTEN expression in the liver macrophages. Consistent with PTEN protein expression, immunofluorescence staining revealed that the PTEN expression was increased in macrophages after APAP treatment (Fig. 2B). Then, we analyzed whether macrophage PTEN deletion may affect the STING-TBK1 pathway in AILI. Unlike the PTEN^FL/FL^ controls, PTEN^M−KO^ reduced p-STING, p-TBK1, p-IRF3, p-P65, RIPK3, and p-MLKL protein expression after APAP treatment livers (Fig. 2C), however, there were no statistical difference in PBS groups (Fig. S1A). IHC staining also revealed decreased RIPK3 expression in the PTEN^M−KO^ livers (Fig. 2D). As compared with PTEN^FL/FL^ controls, the PTEN^M−KO^ livers had reduced mRNA levels coding for tumor necrosis factor-alpha (*Tnf-α*), interleukin-1beta (*IL-1β)*, *IL-6*, C-X-C motif chemokine ligand 1 (*Cxcl-1*), and monocyte chemoattractant protein-1 (*Mcp-1*) (Fig. 2E) following APAP challenge. These data indicate that macrophage PTEN is critical in governing STING-mediated TBK1 activation and RIPK3-mediated necroptosis in AILI.

### Disruption of myeloid-specific PTEN prevents glycogen synthase kinase-3β activation and induces the Notch1/NRF2 pathway in AILI

**Fig. 3:**
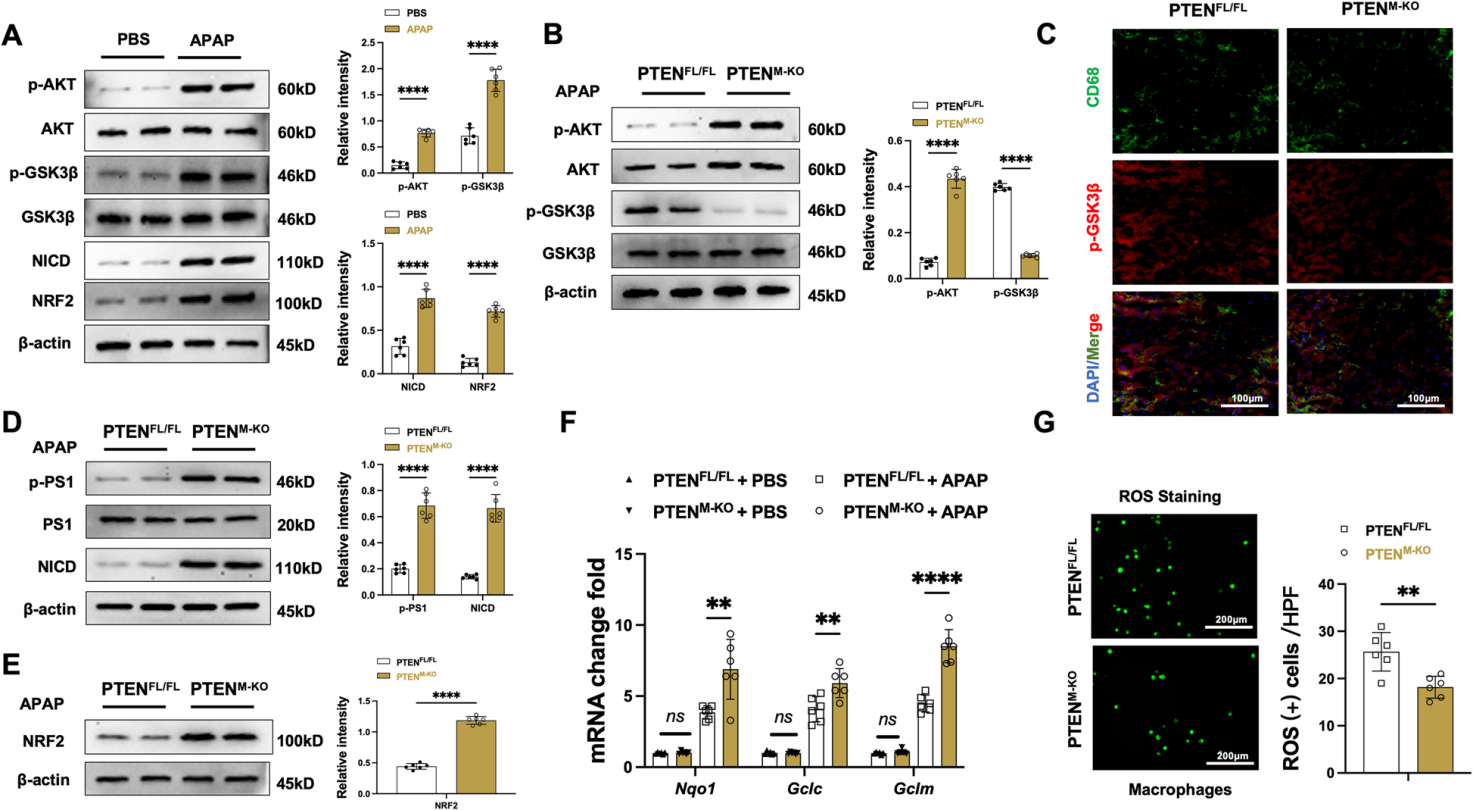
Disruption of myeloid-specific PTEN prevents glycogen synthase kinase-3β (GSK3β) and activates the NICD/NRF2 pathway in AILI. PTEN^FL/FL^ and PTEN^M−KO^ mice were subjected to APAP (400 mg/kg) challenge for 24 h. **A** Western-assisted analysis and relative density ratio of p-Akt, Akt, p-GSK3β, GSK3β, NICD, and NRF2 with or without APAP-challenged PTEN^FL/FL^ livers (*n* = 6 samples/group). **B** Western-blot analysis and relative density ratio of p-Akt, Akt, p-GSK3β, and GSK3β in the PTEN^FL/FL^ and PTEN^M−KO^ mice liver after APAP challenge (*n* = 6 samples/group). **C** Immunofluorescence staining of p-GSK3β and CD68 in AILI liver tissue (*n* = 6 mice/group). Scale bars, 100 μm. **D** Western-blot analysis and relative density ratio of p-PS1, PS1, and NICD in the PTEN^FL/FL^ and PTEN^M−KO^ mice liver after APAP-treated (*n* = 6 samples/group). **E** Western-blot analysis and relative density ratio of NRF2 in the PTEN^FL/FL^ and PTEN^M−KO^ mice livers after APAP-treated. Data representative of three experiments. **F** Quantitative RT-PCR analysis of *Nqo1*, *Gclc*, and *Gclm* mRNA levels in PTEN^FL/FL^ and PTEN^M−KO^ mouse livers with or without APAP-treated (*n* = 6 samples/group). **G** The liver macrophages were isolated from APAP-treated livers, and then these cells were cultured for 2 h at 37℃. Detection of ROS generation by Carboxy-H2DFFDA in LPS-stimulated macrophages from the PTEN^FL/FL^ and PTEN^M−KO^ mice. Quantification of ROS-producing Macrophages (green) (*n* = 6 mice/group). Scale bars, 100 μm. All data represent the mean ± SD. **p < 0.01, ****p < 0.0001

As we have shown that PTEN is involved in STING-TBK1 activation and RIPK3-mediated necroptosis in APAP-indued inflammatory responses, we then explore the potential mechanism of PTEN-mediated inflammation. Our previous study reported that AKT is involved in IR injury as a downstream of PTEN signal [28]; indeed, APAP-induced livers have also been observed to provoke AKT and GSK3β phosphorylation, as well as NICD and NRF2 protein expression (Fig. 3A), compared with the control group. However, PTEN^M−KO^ activated p-Akt and inactivated p-GSK3β in liver tissues after APAP challenge (Fig. 3B). We further examined the expression of p-GSK3β by immunofluorescence staining. Unlike PTEN^FL/FL^ controls, PTEN^M−KO^ decreased p-GSK3β expression in injured livers (Fig. 3C). Previous studies have shown that GSK3β is a negative regulator of Notch activation and stability, GSK3β affects downstream signaling by regulating the phosphorylation of presenilin-1 (PS1), which is the important catalytic subunit of γ-secretase, blocking Notch cleavage [29]. Therefore, we further examined the expression of PS1 and NICD in the injured APAP. Indeed, p-PS1 and NICD expression were increased in the PTEN^M−KO^ livers after APAP treatment (Fig. 3D). Our previous study showed that NRF2 activation relieves stress-induced necroptosis, oxidative stress, and inflammation [22]. Thus, we speculated that PTEN may regulate the NRF2-antioxidative responses in APAP challenges. Notably, PTEN^M−KO^ augmented NRF2 activation (Fig. 3E) and increased antioxidant gene expression coding for NAD(P)H quinone dehydrogenase 1 (*Nqo1*), glutamate-cysteine ligase catalytic subunit (*Gclc*), and glutamate-cysteine ligase regulatory subunit (*Gclm*) (Fig. 3F) in APAP-induced livers. Compared with the PTEN^FL/FL^ controls, ROS production was significantly reduced in the PTEN^M−KO^ macrophages isolated from livers after APAP treatment (Fig. 3G). We also detected the p-AKT, p-GSK3β, p-PS1, NICD and NRF2 expression in PBS groups with PTEN^FL/FL^ and PTEN^M−KO^ mice, but there was no statistical difference (Figure S1B, C, D). Therefore, these data show that macrophage PTEN deficiency leads to p-Akt activation, p-GSK3β inactivation, and NICD/NRF2 activation, resulting in an antioxidative response in APAP-affected livers and reduced damage.

### NRF2 interacts with NICD and mediates STING activation in macrophage

**Fig. 4:**
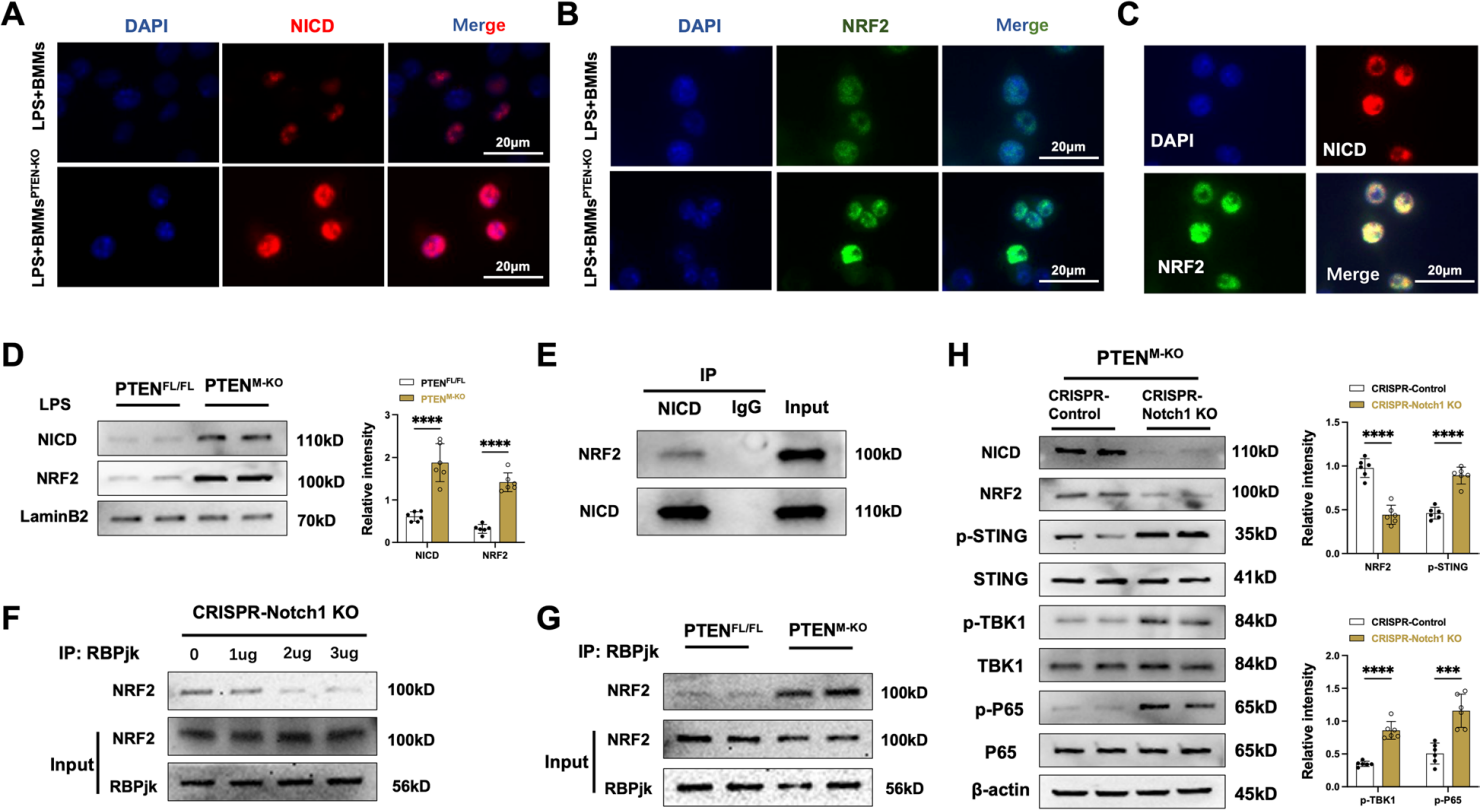
NRF2 interacts with NICD and mediates STING activation in macrophages. Bone marrow-derived macrophages (BMMs, 1 × 10^6^) were isolated from PTEN^M−KO^ and PTEN^FL/FL^ mice and then stimulated with LPS (100 ng/ml) for 6 h. **A** Immunofluorescence staining of NICD (red) in BMMs from PTEN^M−KO^ and PTEN^FL/FL^ after LPS stimulation. Scale bars, 20 μm. Data representative of three experiments. **B** Immunofluorescence staining of NRF2 (green) in BMMs from PTEN^M−KO^ and PTEN^FL/FL^ after LPS stimulation. DAPI was used to visualize nuclei (blue). Scale bars, 20 μm. Data representative of three experiments. **C** Immunofluorescence staining for macrophage NICD (red) and NRF2 (green) in LPS-stimulated macrophages. Scale bars, 20 μm. Data representative of three experiments. **D** Western-blot analysis and relative density ratio of NICD and NRF2 in the PTEN^FL/FL^ and PTEN^M−KO^ macrophage nucleus after LPS stimulation (*n* = 6 samples/group). **E** Immunoprecipitation analysis of NRF2 and NICD in LPS-stimulated macrophages from PTEN^M−KO^ mice. Data representative of three experiments. **F** BMMs were transfected with the CRISPR-Notch1 KO vector (1, 2, and 3ug) or control. Immunoprecipitation analysis of NRF2 and RBPjκ in LPS-stimulated macrophages from PTEN^M−KO^ mice. Data representative of three experiments. **G** Immunoprecipitation analysis of NRF2 and RBPjκ in LPS-stimulated macrophages from PTEN^FL/FL^ and PTEN^M−KO^ mice. Data representative of three experiments. **H** BMMs were transfected with the CRISPR-Notch1 KO vector or control. Western blot analysis and relative density ratio of NICD, NRF2, p-STING, STING, p-TBK1, TBK1, p-P65, and P65 in PTEN^M−KO^ BMMs transfected with the CRISPR-Notch1 KO vector or control after LPS stimulation (*n* = 6 samples/group). All data represent the mean ± SD. ***p < 0.001, ****p < 0.0001

Having demonstrated that macrophage PTEN deficiency promoted NICD and NRF2 activation in the APAP injury livers, we next investigate whether there is a putative molecular mechanism between NICD and NRF2 in the modulation of inflammatory responses. Immunofluorescence staining revealed increased nuclear expression of NICD (Fig. 4A) and NRF2 (Fig. 4B) in LPS-stimulated BMMs. Notably, both NICD and NRF2 (Fig. 4C) were co-localized within the nucleus. To confirm these results, we proceeded to extract nuclear proteins and conduct an analysis of NICD and NRF2 expression in macrophages. As expected, the western blot assay showed that PTEN^M−KO^ macrophages augmented nuclear NICD and NRF2 protein expression after LPS stimulation (Fig. 4D). Next, we used the co-immunoprecipitation (Co-IP) assay to detect the NICD and NRF2 interaction under inflammatory conditions. Indeed, Co-IP obviously revealed that NICD bound to endogenous NRF2 in BMMs after LPS stimulation (Fig. 4E). It is suggested that the interaction between NICD and NRF2 serves a distinct function in the inflammatory response. The NICD translocated to the nucleus, where it interacts with the RBPjκ, and the NICD-RBPjκ complex binds to the Nrf2 promoter region, affecting NRF2 signaling [30]. We then test whether the NICD- RBPjκ interaction could influence RBPjκ-NRF2 binding. Surprisingly, CRISPR-mediated Notch1 KO reduced nuclear RBPjκ-NRF2 binding (Fig. 4F) in BMMs, whereas PTEN^M−KO^ macrophages augmented RBPjκ-NRF2 binding after LPS stimulation (Fig. 4G). The data indicate that the translocation of NICD to the nucleus has the potential to govern RBPjκ-NRF2 binding, thereby exerting control over NRF2 activity. We further utilized CRISPR-mediated Notch1 KO macrophages to demonstrate that NICD and NRF2 binding is necessary for PTEN to trigger STING-mediated innate immune responses in vitro. As expected, the deletion of Notch1 reduced NRF2 expression but increased p-STING, p-TBK1, and p-P65 protein expression (Fig. 4H) in macrophages from PTEN^M−KO^ mice. The study suggests that NRF2 may interact with NICD through RBPjκ in the macrophage nucleus to modulate PTEN control of the STING-mediated innate immune response.

### NRF2 is vital for inhibiting STING/TBK1 activation in macrophage PTEN‑mediated inflammatory response in response to AILI

**Fig. 5:**
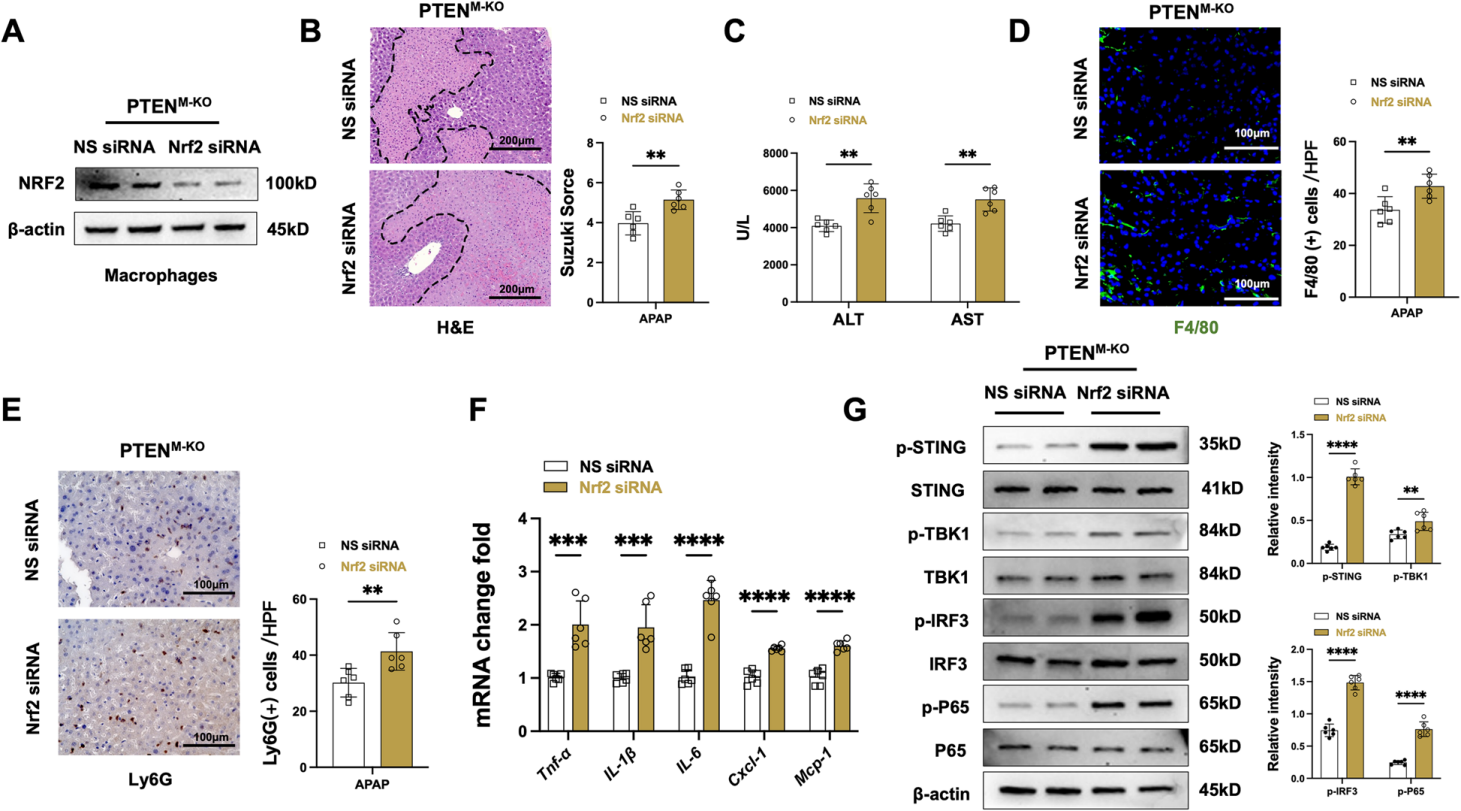
NRF2 is vital for  inhibiting STING/TBK1 activation in  macrophage PTEN‑mediated inflammatory in response to AILI. The PTEN^M−KO^ mice were injected via tail vein with Nrf2 siRNA (2 mg/kg) or non-specific (NS) control siRNA mixed with mannose-conjugated polymers at 24 h prior to APAP (400 mg/kg) treatment. **A** Western blot confirmed NRF2 expression with liver macrophages isolated for PTEN^M−KO^ mice. Data representative of three experiments. **B** Representative histological staining (H&E) of APAP-conditioning liver and Suzuki’s histological score (*n* = 6 samples/group). Scale bars, 200 μm. **C** Liver function was measured by serum ALT and AST levels (IU/L) (*n* = 6 samples/group). **D** Immunofluorescence staining of F4/80 macrophages in injured livers (*n* = 6 mice/group). Quantification of F4/80^+^ macrophages. Scale bars, 100 μm. **E** Immunohistochemistry staining of Ly6G neutrophils in injured livers (*n* = 6 mice/group). Quantification of Ly6G^+^ neutrophils. Scale bars, 100 μm. **F** Quantitative RT-PCR-assisted detection of *Tnf-α*, *IL-1β*, *IL-6*, *Cxcl-1,* and *Mcp-1* mRNA levels in injured livers (*n* = 6 samples/group). **G** Western-assisted analysis and relative density ratio of p-STING, STING, p-TBK1, TBK1, p-IRF3, IRF3, p-P65, and P65 in injured livers. All data represent the mean ± SD. **p < 0.01, ***p < 0.001, ****p < 0.0001

Having demonstrated the interaction of NICD and NRF2 in PTEN^M−KO^ macrophages, we dissect the role of NFR2 in macrophage PTEN-mediated immune moderation in APAP-triggered liver. We disrupted NFR2 in PTEN^M−KO^ livers with an in vivo mannose-mediated Nrf2 siRNA delivery system specifically delivered to macrophages by expressing a mannose-specific membrane receptor [26]. Firstly, the Nrf2 siRNA treatment effectively eliminated NRF2 expression in isolated liver macrophages obtained from PTEN^M−KO^ mice (Fig. 5A). Knockdown of Nrf2 with the mannose-mediated siRNA treatment aggravated APAP-challenged liver injury, as evidenced by increased Suzuki’s histological score (Fig. 5B) and sALT and sAST levels (Fig. 5C), compared to the control group. Then, we investigated the inflammatory cell infiltration in livers; Nrf2 siRNA treatment increased F4/80^+^ macrophage (Fig. 5D) and Ly6G^+^ neutrophil (Fig. 5E) accumulation in the PTEN^M−KO^ APAP-treated livers. Similarly, the mRNA levels of *Tnf-α*, *IL-1β*, *IL-6*, *Cxcl-1*, and *Mcp-1* in APAP-challenged livers were also augmented after the knockdown of Nrf2 (Fig. 5F). Lastly, unlike the control group, depleted Nrf2 increased p-STING, p-TBK1, p-IRF3 and p-P65 expression (Fig. 5G) in the PTEN^M−KO^ livers. These results elucidate that NRF2 is a critical regulator of PTEN control STING-mediated inflammatory response in AILI.

### STING plays a critical role in the PTEN‑mediated inflammatory response and ROS generation in LPS‑stimulated macrophages

**Fig. 6:**
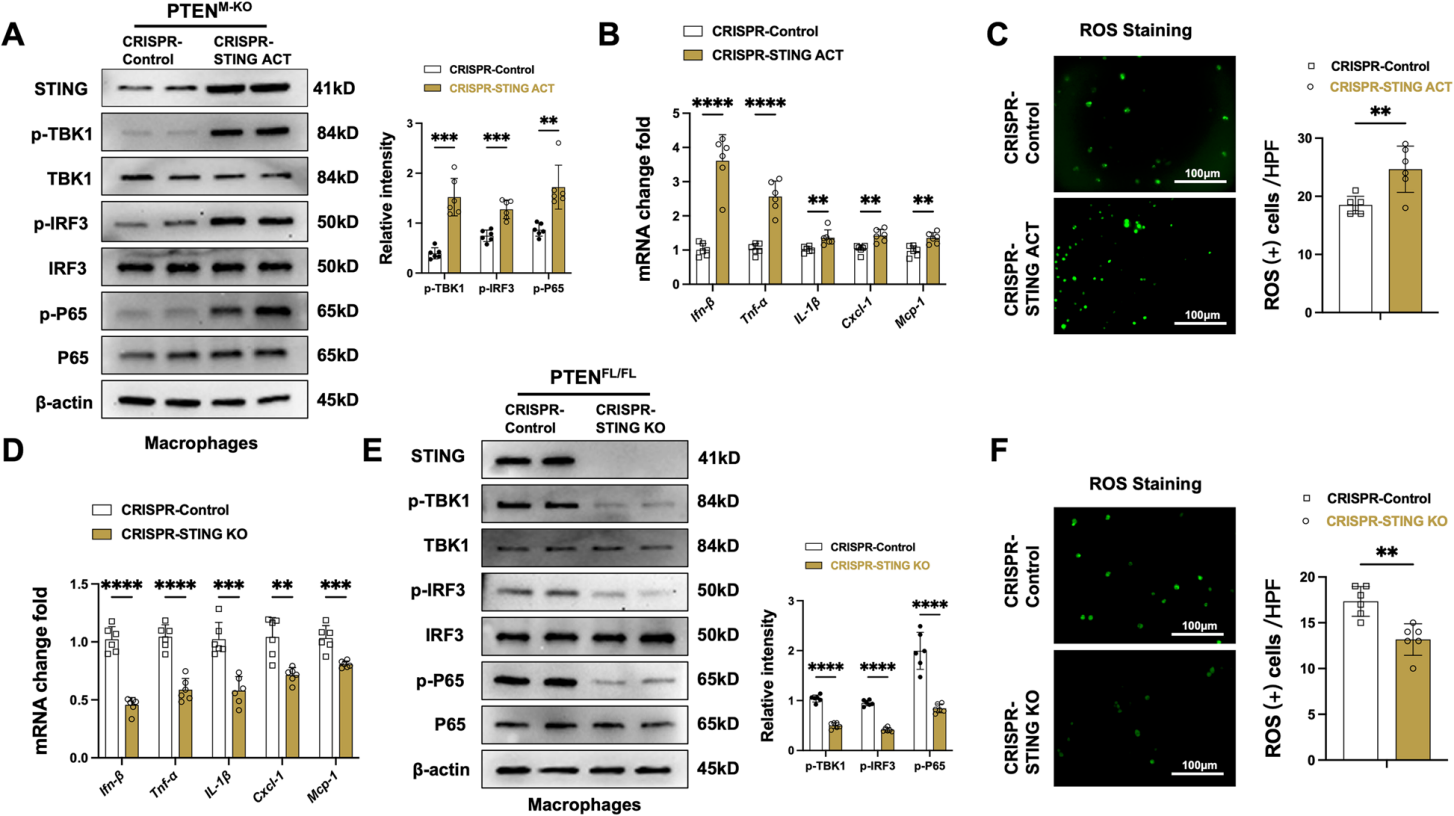
STING plays a critical role in the PTEN‑mediated inflammatory response and ROS generation in LPS‑stimulated macrophages. Bone marrow-derived macrophages (BMMs) were isolated from PTEN^M−KO^ or PTEN^FL/FL^ mice and then transfected with CRISPR/Cas9-mediated STING activation (ACT) or STING knockout (KO) vector. **A** Western blot analysis and relative density ratio of STING, p-TBK1, TBK1, p-P65, and P65 in LPS-stimulated BMMs transfected with CRISPR/Cas9-mediated STING ACT and control from PTEN^M−KO^ mice (*n* = 6 samples/group). **B** Quantitative RT-PCR analysis of *Ifn-β*, *Tnf-α*, *IL-1β*, *Cxcl-1,* and *Mcp-1* mRNA levels in LPS-stimulated BMMs transfected with CRISPR/Cas9-mediated STING ACT and control from PTEN^M−KO^ mice (*n* = 6 samples/group). (C) Detection of ROS generation by Carboxy-H2DFFDA in LPS-stimulated BMMs transfected with CRISPR/Cas9-mediated STING ACT and control from PTEN^M−KO^ mice. Quantification of ROS-producing macrophages (green) (*n* = 6 samples/group). Scale bars, 100 μm. **D** Quantitative RT-PCR analysis of *Ifn-β*, *Tnf-α*, *IL-1β*, *Cxcl-1,* and *Mcp-1* mRNA levels in LPS-stimulated BMMs transfected with CRISPR/Cas9-mediated STING KO and control from PTEN^FL/FL^ mice (*n* = 6 samples/group). **E** Western blot analysis and relative density ratio of STING, p-TBK1, TBK1, p-P65, and P65 in LPS-stimulated BMMs transfected with CRISPR/Cas9-mediated STING KO and control from PTEN^FL/FL^ mice (*n* = 6 samples/group). **F** Detection of ROS generation by Carboxy-H2DFFDA in LPS-stimulated BMMs transfected with CRISPR/Cas9-mediated STING KO and control from PTEN^FL/FL^ mice. Quantification of ROS-producing macrophages (green) (*n* = 6 samples/group). Scale bars, 100 μm. All data represent the mean ± SD. **p < 0.01, ***p < 0.001, ****p < 0.0001

To explore the putative mechanism of the STING in the modulation of PTEN-mediated inflammation and ROS generation in macrophages after APAP-challenged livers, we used BMMs from PTEN^M−KO^ and PTEN^FL/FL^ mice transfected with CRISPR/Cas9-mediated STING activation or STING KO vector. CRISPR/Cas9-mediated STING activation augmented p-TBK1, p-IRF3 and p-P65 protein expression (Fig. 6A), as well as enhanced mRNA levels of interferon-β (*Ifn-β)*, *Tnf-α*, *IL-1β*, *Cxcl-1,* and *Mcp-1* (Fig. 6B) in PTEN^M−KO^ macrophages after LPS-stimulation. Carboxy-H2DFFDA indicated increased ROS production in CRISPR-STING activation macrophages (Fig. 6C). On the contrary, we used BMMs transfected with a CRISPR/Cas9-mediated STING KO vector with PTEN^FL/FL^ mice. The results showed that the *Ifn-β*, *Tnf-α*, *IL-1β*, *Cxcl-1,* and *Mcp-1* mRNA coded declined (Fig. 6D), along with the p-TBK1, p-IRF3 and p-P65 protein expression (Fig. 6E). Meanwhile, ROS generation was also down-regulated (Fig. 6F) in PTEN^FL/FL^ mice macrophages after LPS stimulation. The data suggests that the STING pathway plays a substantial role in both PTEN-driven immune modulation and reactive oxygen species production.

### Macrophage PTEN deficiency‑mediated STING enhances stress‑induced hepatocyte necroptosis via modulating RIPK3 signaling activation

**Fig. 7:**
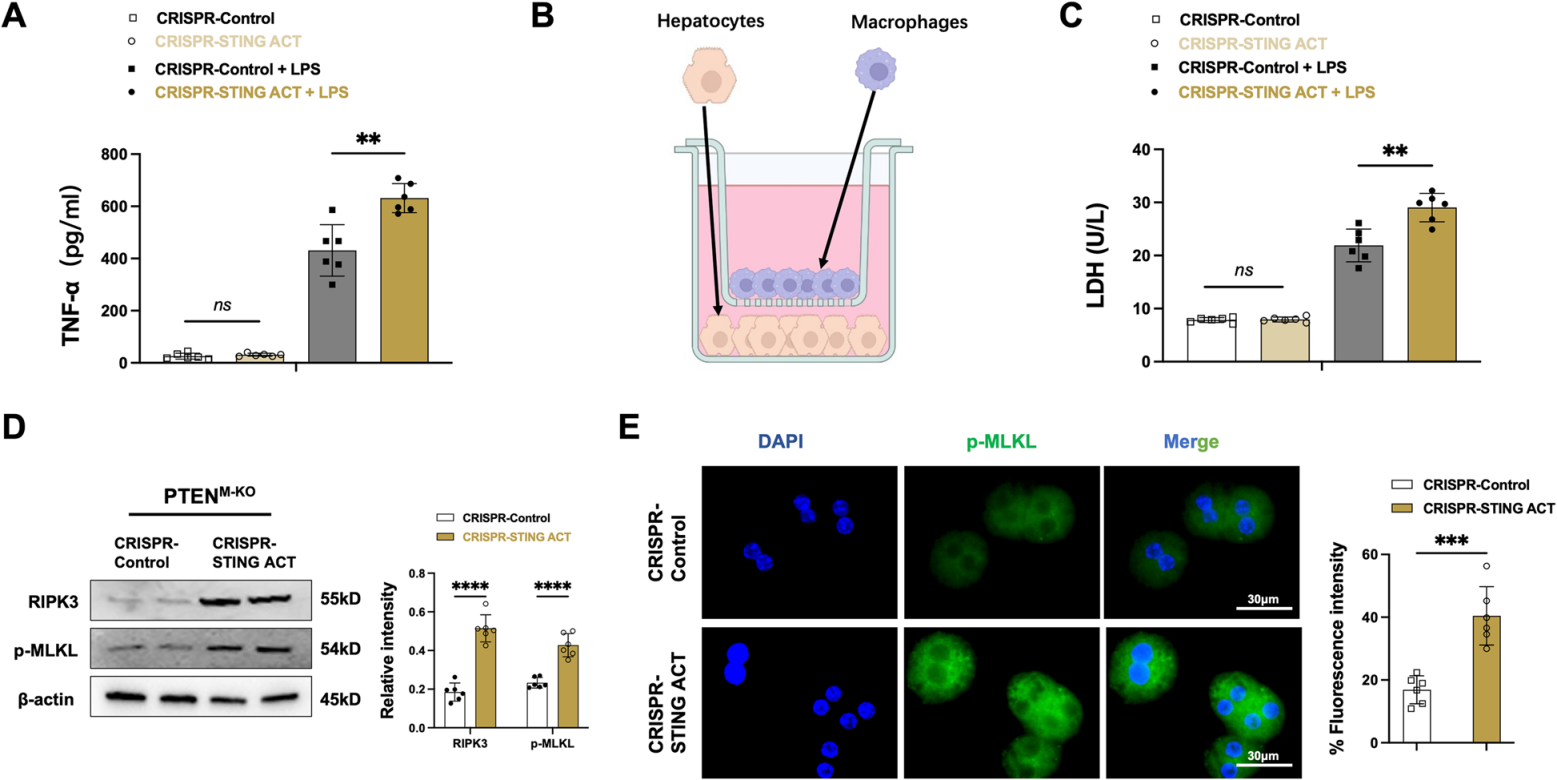
Macrophage PTEN deficiency‑mediated STING enhances stress‑induced hepatocyte necroptosis via modulating RIPK3 signaling activation. Bone marrow-derived macrophages (BMMs) were isolated from PTEN^M−KO^ mice and then transfected with CRISPR/Cas9-mediated STING ACT and control vector. **A** ELISA analysis of supernatant TNF-α levels with or without LPS-stimulated BMMs (*n* = 6 samples/group). **B** The figure for macrophage/hepatocyte co-culture system. BMMs transfected with CRISPR/Cas9-mediated ACT were stimulated with LPS 6 h and then cocultured with primary hepatocytes supplemented with H_2_O_2_ for 24 h. **C** ELISA analysis of LDH release from hepatocytes in cocultures (*n* = 6 samples/group). **D** Western-blot analysis and relative density ratio of RIPK3 and p-MLKL in hepatocytes after coculture (*n* = 6 samples/group). **E** Immunofluorescence staining and fluorescence intensity analysis of p-MLKL^+^ hepatocytes after co-culture (*n* = 6 samples/group). Scale bars, 30 μm. All data represent the mean ± SD. *p < 0.05. **p < 0.01, ***p < 0.001

We lastly elucidate how macrophage STING may regulate hepatocyte necroptosis during the inflammatory and stress response. As oxidative stress induces TNF-α production, which plays a dynamic role in cell death [31]. Using the enzyme-linked immunosorbent assay (ELISA), TNF-α release from LPS-stimulated PTEN^M−KO^ BMMs after transfection with a CRISPR-STING activation vector was augmented (Fig. 7A), compared to the control group. Using a co-culture system with primary hepatocytes treated with H_2_O_2_ and with or without LPS-stimulated CRISPR-STING activation BMMs from the PTEN^M−KO^ mice (Fig. 7B), we found that the LDH released (Fig. 7C) from oxidative stressed hepatocytes was markedly augmented, and hepatocyte RIPK3 and p-MLKL expression (Fig. 7D) were significantly enhanced in the macrophage STING activation group with LPS-stimulated. Accordingly, compared to the control group, we found that immunofluorescence staining showed increased p-MLKL^+^ hepatocytes in the CRISPR-STING activation BMMs co-culture system (Fig. 7E). The findings suggest that a deficiency in PTEN within the macrophages, coupled with STING overexpression, leads to an increase in the release of TNF-α, which subsequently results in heightened hepatocyte necroptosis by amplifying the signaling of RIPK3-p-MLKL in hepatocytes.

## Discussion

This study for the first-time documents that macrophage PTEN is crucial for orchestrating inflammatory responses, NICD and NRF2 signaling interaction, and STING activation in liver inflammatory injury due to APAP challenges. In detail, the principal findings of this study are as follows: i) macrophage PTEN deficiency alleviates APAP-induced liver damage, reduces macrophage/neutrophil trafficking and proinflammatory mediators; ii) macrophage PTEN deficiency improves ROS generations and moderates APAP-triggered hepatocellular necroptosis; iii) macrophage PTEN deficiency inhibited STING-mediated inflammatory responses and regulates hepatocyte necroptosis via NICD and NRF2 crosstalk. Thus, these findings highlight the crucial role of macrophage PTEN in regulating innate immune responses in AILI.

PTEN plays a pivotal role in controlling intracellular signaling for cell survival and proliferation by regulating the PI3K/Akt pathway [32], which is involved in many diseases, including hepatocellular carcinoma [33], liver fibrosis [34], liver ischemia and reperfusion injury [35] and DNA damage [36]. Consistent with the role of PTEN-PI3K/Akt in the inflammation cascade, our current in vivo study has shown that myeloid-specific PTEN knockout ameliorates AILI, evidenced by decreased sALT levels, histological liver damage, local macrophage/neutrophil accumulation, proinflammatory cytokine gene expression, and hepatocellular death, concomitant activation of AKT phosphorylation. This is consistent with the previous finding that macrophage PTEN deficiency promoted cytoprotection in other liver inflammations induced by ischemia and reperfusion [35].

As a putative regulator of oxidative stress, GSK3β is a downstream target gene of Akt, which is seen to be specifically reliant on Akt phosphorylated at Ser473 residues [30]. It has also been highlighted how GSK3β and NRF2 activation is important. NRF2 is a transcription factor that modulates the cellular defense against oxidative stress via activating antioxidant genes. When the cell is under stress, NRF2 is transported to the nucleus, which triggers the transcription of the genes that are its downstream targets [37]. Growing research suggests that GSK3β promotes NRF2 degradation without Keap1 by phosphorylating Ser residues in the Neh6 domain [38]. Our previous studies demonstrated that NRF2 improved IR stress-induced liver damage via antioxidant responses [22]. Furthermore, disruption of NRF2 signaling could increase ROS generation and inflammatory response [39]. Consistent with prior findings, we found that PTEN^M−KO^ decreased GSK3β phosphorylation and thus inhibited NRF2 degradation in the cytoplasm, enhanced nuclear NRF2 activity, and eventually led to reduced ROS generation, indicating that NRF2 could play an essential role in macrophage PTEN-mediated immune regulation.

The initial stage of Notch signaling involves a sequence of cleavage events, with the second step relying on γ-secretase activity. This event results in the formation of the NICD, which subsequently translocates to the nucleus to activate Notch target genes. The deregulation of the Notch cascade has been identified as a significant contributing factor to numerous pathological conditions. For example, our previous study has shown that in TLR-activated macrophages, the NICD promotes the production of the anti-inflammatory regulator, reducing the release of proinflammatory cytokines [16]. Further research found that deletion of myeloid Notch1 activity triggers RhoA/ROCK signaling and aggravates liver inflammation [28]. The cleavage of Notch requires the participation of γ-secretase, an enzyme made up of four subunits: the catalytic subunit PS1 or PS2, Pen-2, APH-1, and nicastrin (NCT) [29]. Given this, it can be concluded that secretase is essential for the stability and function of Notch. γ-secretase inhibitors have undergone development and testing for their efficacy in treating cancers that are associated with Notch, including T-cell acute lymphoblastic leukemia, as well as for the treatment of Alzheimer’s disease [40]. It has been reported that PS-1 is targeted by GSK3β. The loop domain of presenilin-1 is composed of conserved GSK3β phosphorylation sites, with negative regulation of the PS-1 pool occurring via GSK3β phosphorylation [41]. It is plausible that GSK3β plays a role in managing the cleavage of the Notch, thereby initiating the Notch signaling pathway downstream. This study characterizes that GSK3β not only regulates NRF2 activity but also simultaneously regulates Notch cleaving during AILI. We found that PTEN^M−KO^ inhibited GSK3β phosphorylation and activated Notch1 cleavage via PS-1 phosphorylation, resulting in increased NICD entry into the nucleus during APAP challenges.

We then need to investigate how PTEN can regulate the nuclear translocation of the NICD and NRF2 signals in the liver during APAP stress. We speculate that nuclear localization of endogenous NICD and NRF2 may be essential for the modulation of STING/TBK1 activation in APAP-stressed livers. Previous studies have shown that NRF2 can regulate Notch signaling in liver regeneration models [42]. Indeed, using an in vitro culture system, we discovered that macrophage NRF2 and NICD co-localized in the nucleus and increased nuclear expression of NRF2 and NICD in response to LPS stimulation. Moreover, NRF2 interacted with NICD via RBPjκ. Knockout Notch1 expression reduced nucleus RBPjκ-NRF2 binding, suggesting a possible regulatory mechanism in which NICD- RBPjκ-NRF2 complex controls NRF2 activity. On the contrary, the PTEN^M−KO^ macrophages increased the NICD expression argument of the RBPjκ-NRF2 binding and enhanced NRF2 activity. In addition, Notch1 depletion reduces NRF2 expression and increases STING-mediated inflammation in LPS-stimulated macrophages. Our data highlight a new function of macrophage PTEN signaling in controlling the inflammatory response driven by STING-TKB1 during liver injury caused by APAP. In line with our in vivo findings, we showed that NRF2 siRNA aggravated AILI and increased F4/80^+^ macrophage and Ly6G^+^ neutrophil accumulation, and exacerbated proinflammatory cytokine, in proportion to activated STING-TBK1 signaling in liver tissue. Our research has uncovered a surprising function of macrophage PTEN signaling in regulating the NICD/NRF2 pathway, which controls the STING-mediated immune response during liver sterile inflammation triggered by APAP.

In addition, macrophage PTEN-mediated STING signaling may regulate APAP-induced necroptosis pathways. As we know, ROS can induce oxidative mitochondrial damage, leading to mtDNA leaks into the cytoplasm [18]. Activated by cytosolic DNA, the cyclic GMP-AMP synthase (cGAS)-STING pathway is a critical innate immune system signaling cascade [18]. Numerous liver diseases, such as viral hepatitis, non-alcoholic fatty liver disease, alcoholic liver disease, primary hepatocellular carcinoma, and hepatic ischemia–reperfusion damage, have been linked to the crucial function of cGAS-STING signaling [43]. cGAMP binds and activates the adaptor protein STING to induce IRF3 activation via TBK1 [44]. Previous studies reveal that the STING-IRF3 pathway induces inflammation and apoptosis, disrupting glucose and lipid metabolism in hepatocytes [45]. Aged mice with disruption of STING were protected from sterile inflammatory liver damage [46]. Compared with wild-type mice, STING-deficient mice presented decreased IR-induced liver injury [47]. These data suggest that STING signaling plays a critical role in liver injury. Furthermore, NICD interacted with STING at the CDN binding domain, preventing the cGAS binding to STING and obstructing the activation of STING signaling [48], which is in accordance with the fact that Nocth1 knockout could promote p-STING expression in this document. Next, we employed CRISPR/Cas9-mediated STING ACT to augment p-TBK1, p-IRF3, and p-P65 expression, elevate pro-inflammatory gene coding, and amplify ROS generation in macrophages of PTEN^M−KO^ mice. The opposite occurs in PTEN^FL/FL^ mouse macrophages using the CRISPR/Cas9-mediated STING KO plasmid. The generation of ROS instigates necroptosis, a cellular process that necessitates the participation of RIPK3 and its substrate, MLKL. RIPK3 activation, which is controlled by caspase-mediated cleavage and serves as a crucial switch for necroptosis and inflammation, enhanced inflammation-related cell death and damage in several inflammatory disease models [49]. Phosphorylation-mediated activation of MLKL causes it to move to the inner leaflet of the plasma membrane, disrupting cell integrity and prompting necroptosis [50]. Likewise, our previous insightful work demonstrated that RIPK3 disruption decreased caspase-1 activity by impeding NEK7/NLRP3 activity in hepatic ischemia–reperfusion damage [26]. The current work, using a macrophage/hepatocyte co-culture system in vitro, demonstrated that STING ACT-mediated CRISPR/Cas9 promotes TNF-α release in PTEN^M−KO^ macrophages stimulated by LPS. We revealed that macrophage STING overexpression elevated the expression of p-MLKL and RIPK3 on hepatocytes and accelerated LDH release from hepatocytes that had been exposed to H_2_O_2_. Additionally, the results of immunofluorescence imaging confirmed that macrophage STING upregulation enhanced hepatocyte necroptosis in H_2_O_2_-induced damage. These results indicate that STING signaling is key to controlling AILI in macrophage PTEN-mediated innate immune responses.

Due to the complex nature of the identified regulatory process, our study has several limitations. Firstly, to generate myeloid-specific PTEN knockout mice, we used Lys2 (LysM)- cre mouse mated with loxP-flanked PTEN mouse. However, while myeloid cells, in their majority: macrophages, monocytes, and granulocytes, originate from the same linage, we haven’t assessed the efficacy of PTEN deletion in neutrophils. Secondly, recent single-cell and spatial transcriptomic technologies have revealed an underappreciated heterogeneity of liver macrophages in liver disease. To represent macrophages, we exclusively utilized F4/80 or CD68 without additional specification of distinct macrophage subtypes. Finally, our co-culture experiments provide insight into the communication between macrophages and hepatocytes. However, the exact nature of this interaction requires further investigation. Exploring the signaling pathways and molecules involved in this crosstalk would be beneficial to better understand the mechanisms underlying our observations.

## Conclusions

In this study, we demonstrated that macrophage PTEN regulates STING activation through NICD and NRF2 signaling crosstalk in AILI. Disruption of macrophage PTEN activates PI3K/Akt, inactivates GSK3β, activates NICD/NRF2, and ultimately inhibits STING-TBK1 signaling, reducing APAP-induced liver inflammation. Our results highlight the critical role of the macrophage PTEN-NICD/NRF2-STING axis in regulating the innate immune response in AILI and suggest that it has therapeutic potential for managing sterile liver inflammation.

## Supplementary Information

**Additional file** **1:** **Table 1.** Primer sequences for the amplification

**Additional file** **2:** **Supplemental Figure 1. **The protein expression in liver tissue from PTEN^FL/FL^ and Notch1^M-KO^ mice after PBS injection.

## Data Availability

The data used to support the findings of this study are included in the article.
