# Supplementary material for: Macrophage PTEN controls STING-induced inflammation and necroptosis through NICD/NRF2 signaling in APAP-induced liver injury
Source: Cell Commun Signal. 2023 Jun 27;21:160. doi: 10.1186/s12964-023-01175-4 (PMC10294406; doi:10.1186/s12964-023-01175-4)
Supplement: Supplementary file 2 — Additional file 1: Table 1. Primer sequences for the amplification [file 12964_2023_1175_MOESM1_ESM.docx]

| **Table 1**: Primer sequences for the amplification | | |
| --- | --- | --- |
| **Target genes** | **Forward primers** | **Reverse primers** |
| ***mβ-Actin*** | 5’- GTGACGTTGACATCCGTAAAGA-3’ | 5’- GCCGGACTCATCGTACTCC-3’ |
| ***mTnf-α*** | 5’- ACGGCATGGATCTCAAAGAC-3’ | 5’- AGATAGCAAATCGGCTGACG-3’ |
| ***mIfn-β*** | 5’-CCACTTGAAGAGCTATTACTG-3’ | 5’- AATGATGAGAAAGTTCCTGAAG-3’ |
| ***mIL-1β*** | 5’- TGTAATGAAAGACGGCACACC-3’ | 5’- TCTTCTTTGGGTATTGCTTGG-3’ |
| ***mIL-6*** | 5’- GCTACCAAACTGGATATAATCAGGA -3’ | 5’-CCAGGTAGCTATGGTACTCCAGAA -3’ |
| ***mMcp-1*** | 5'-GAAGGAATGGGTCCAGACAT-3' | 5'-ACGGGTCAACTTCACATTCA-3' |
| ***mCxcl-1*** | 5’-TGGCTGGGATTCACCTCAAGAACA-3’ | 5’-TTTCTGAACCAAGGGAGCTTCAGG-3’ |
| **mNqo1** | 5’-AGCTGGAAGCTGCAGACCTG-3’ | 5’-CCTTTCAGAATGGCTGGCA-3’ |
| ***mGclc*** | 5’-ATCTGCAAAGGCGGCAAC-3’ | 5’-ACTCCTCTGCAGCTGGCTC-3’ |
| ***mGclm*** | 5’-TGGAGCAGCTGTATCAGTGG-3’ | 5’-AGAGCAGTTCTTTCGGGTCA -3’ |
