# Supplementary material for: Macrophage PTEN controls STING-induced inflammation and necroptosis through NICD/NRF2 signaling in APAP-induced liver injury
Source: Cell Commun Signal. 2023 Jun 27;21:160. doi: 10.1186/s12964-023-01175-4 (PMC10294406; doi:10.1186/s12964-023-01175-4)
Supplement: Supplementary file 3 — Additional file 2: Supplemental Figure 1. The protein expression in liver tissue from PTENFL/FL and Notch1M-KO mice after PBS injection. [file 12964_2023_1175_MOESM2_ESM.docx]

**Supplementary Materials**

**Macrophage PTEN controls STING-induced** **inflammation and necroptosis through** **NCID/NRF2 signaling in APAP-induced liver injury**

**Tao Yang^1,3#^, Xiaoye Qu^2#^, Jiaying Zhao^1#^, Xiao Wang^1#^**, Qian Wang^1^, Jingjing Dai^1^, Chuanlong Zhu^1^, Jun Li^1*^, Longfeng Jiang^1*^

^1^Department of Infectious Diseases, The First Affiliated Hospital with Nanjing Medical University, Nanjing, China

^2^Department of Liver Surgery, Renji Hospital, Shanghai Jiaotong University School of Medicine, Shanghai, China

^3^Department of Respiratory and Critical Care Medicine, The Afﬁliated People’s Hospital of Jiangsu University, The Zhenjiang Clinical Medical College of Nanjing Medical University, Zhenjiang, China;

**^#^These authors contributed equally to this work**

**Supplemental Figure**

**Figure S1**

**
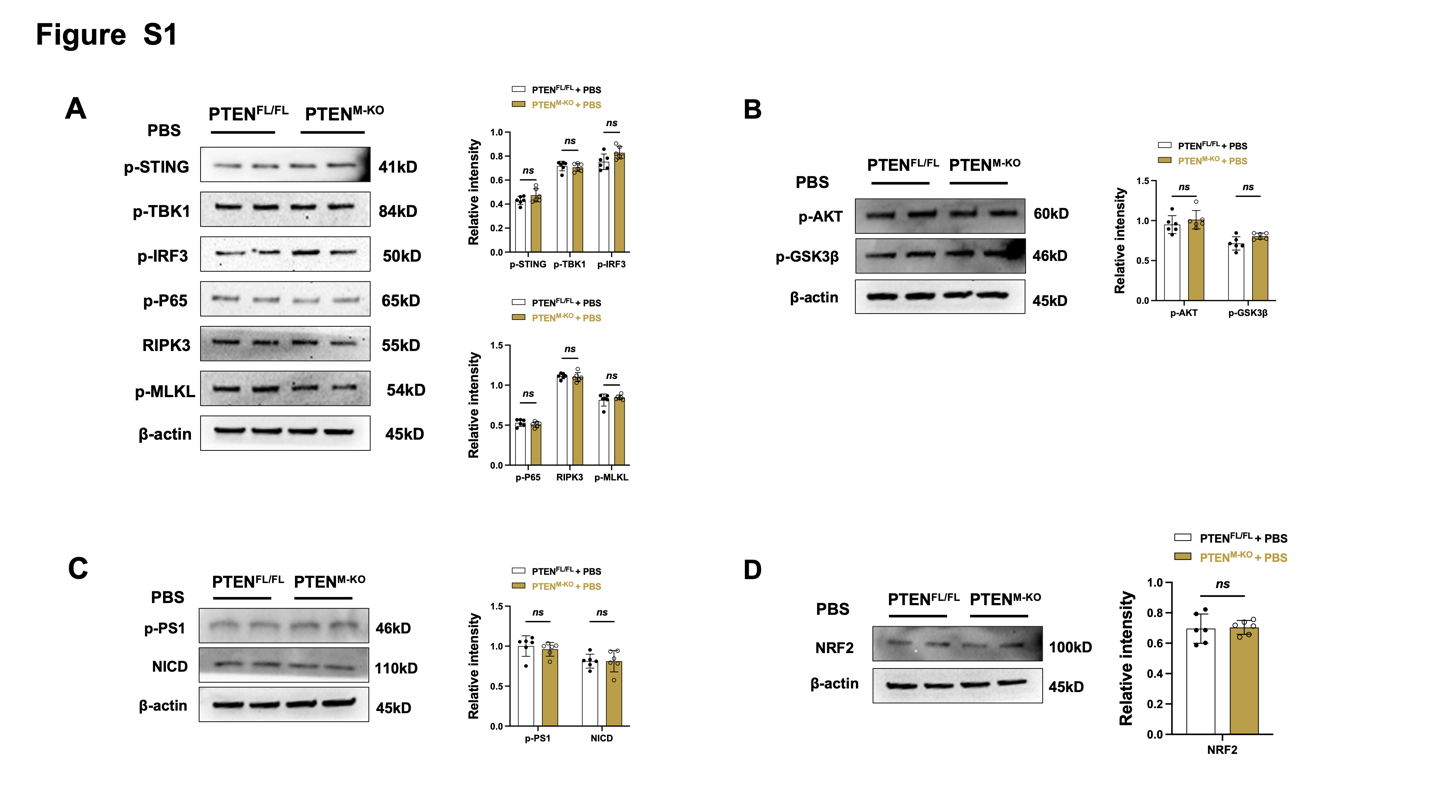
**

**Supplemental figure 1.** **The protein expression in liver tissue from** **PTEN^FL/FL^ and Notch1^M-KO^ mice after PBS injection.**

(A) Western blot analysis and relative density ratio of p-STING, p-TBK1, p-IRF3, p-P65, RIPK3, and p-MLKL (n=6 samples/group). (B) Western blot analysis and relative density ratio of p-AKT and p-GSK3β (n=6 samples/group). (C) Western blot analysis and relative density ratio of p-PS1 and NICD (n=6 samples/group). (B) Western blot analysis and relative density ratio of NRF2 (n=6 samples/group). All data represent the mean*±* SD.
